# Novel halos in light kaonic nuclei as an indicator of nuclear equation of state at supra-normal densities

**DOI:** 10.1038/s41598-017-16877-2

**Published:** 2017-12-01

**Authors:** Rong-Yao Yang, Wei-Zhou Jiang, Si-Na Wei, Dong-Rui Zhang

**Affiliations:** Department of Physics, Southeast University, Nanjing, 211189 China

## Abstract

The sensitive correlations between the low-density halo structure and the high-density properties of the nuclear equation of state (EOS) are constructed in light kaonic nuclei with the relativistic mean-field theory. More specifically, the 1*p*_1/2_ halo spreads out linearly with increasing the pressure and sound velocity square at supra-normal densities and decreasing the incompressibility at saturation density. These results suggest that the novel halo in light kaonic nuclei can serve as a sensitive indicator of the nuclear EOS of symmetric matter at supra-normal densities. The experimental production and detection of the light kaonic nuclei, yet to be available, is discussed in some details at last.

## Introduction

The nuclear equation of state (EOS) plays a crucial role in nuclear structures, reaction dynamics and many issues in astrophysics. As a residual interaction of the quantum chromodynamics, the nuclear force and the resulting EOS have not been well determined in the medium especially at supra-normal densities due to the complexity of the many-body problem. Even with the nuclear potentials that fit the deuteron properties and nucleon-nucleon scattering data, the nuclear saturation in microscopic approaches does not turn out to be straightforward^1–3^. Moreover, theoretical models that fit the properties of nuclear saturation and finite nuclei yield a large variety of symmetric matter EOS’s^4–6^ and various density dependence of symmetry energy^7–9^ both of which differ largely at supra-normal densities. In terrestrial laboratories^9–14^, the energetic heavy-ion reactions are currently the unique way to determine the high-density EOS, while the celestial observation of neutron stars (NS’s) may provide another hopeful way to constrain the high-density EOS^15–22^. However, the extracted EOS suffers from large uncertainties that are close to a large relative error of 50% at high densities^9,11,19^. Moreover, significant uncertainties of the celestial constraints may also arise from the possible dark matter contamination in NS’s^23,24^ and potential deviation from the standard Einstein theory in strong-field limits, e.g., see ref.^25^. Although the elementary forces and the structure of matter are two basic subjects in physics, such a large systematic error prohibits indeed from extracting structural properties such as phase transitions and matter constituents. Thus, it is of prime importance and broad interest to pursuit accurate extraction of the nuclear EOS at supra-normal densities.

It is well known that the structural properties of finite nuclear system may accurately constrain the nuclear forces and the resulting nuclear EOS near or beneath saturation density. Recently, the properties of exotic nuclei have played a special role in constraining the nuclear forces. For instance, the exotic states, such as the halo^26–28^ and Hoyle states^29,30^, and the shell evolution anomaly^31,32^ reveal the importance of the three-body force, while the novel magic numbers far off the *β*-stability are mainly subject to the role of the tensor force^33–35^. It is, however, unfortunate that the high-density EOS can not be determined by the structural properties of finite nuclei, since the extrapolation of the EOS’s to high densities varies greatly. Thus, novel systems that feature a much compacter core should be created to constrain the high-density EOS directly. While it is impossible to acquire a clear rise of the core density by adding more nucleons in finite nuclei, the inclusion of the new degree of freedom such as the strangeness becomes a uniquely possible way to realize a much denser core in finite nuclei because of the additional attraction. Typical examples are the metastable exotic multihypernuclear objects^36–38^ and kaonic nuclei^39–41^. In particular, due to the strongly attractive interaction between nucleons and *K* ^−^ meson, the deep *K* ^−^-nuclear bound states may form, resulting in a high-density core in light kaonic nuclei^40–45^. Up to now, continuous experimental efforts have been made progressively to search for kaonic nuclei^46–54^. In this paper, we propose, for the first time, that the strangeness in deeply bound kaonic nuclei may provide a novel mechanism for the formation of the exotic structure, the diffusive nuclear halo. This brand-new mechanism enables the halo formation in nuclei of *β*-stability, while the normal halos are usually restrained to the neighborhood of the drip lines^55–57^. We will find that the property of the low-density halo correlates sensitively with the incompressibility at saturation density and the pressure and sound velocity at supra-normal densities. These relationships arising from the different density regimes in the same system will provide accurate constraints on the high-density EOS.

## Formalism

In this work, the light kaonic nuclei are studied in the relativistic mean-field (RMF) models^58–61^. We adopt the RMF model to study the deeply-bound kaonic nuclei not only because it reproduces nicely the ground-state properties for nuclei in the whole nuclide table, but also the RMF approximation was originally made to fit denser matter where the intermediate-state contribution is more suppressed by the Pauli blocking^58,60^. The interacting Lagrangian is written as^62,63^

1 $$\begin{array}{rcl}{{ {\mathcal L} }}_{int} & = & {\bar{\psi }}_{N}[{g}_{\sigma N}\sigma -{g}_{\omega N}{\gamma }_{\mu }{\omega }^{\mu }-{g}_{\rho N}{\gamma }_{\mu }{\tau }_{3}{b}_{0}^{\mu }-e\frac{1+{\tau }_{3}}{2}{\gamma }_{\mu }{A}^{\mu }]{\psi }_{N}\\  &  & -\frac{1}{3}{g}_{2}{\sigma }^{3}-\frac{1}{4}{g}_{3}{\sigma }^{4}+\frac{1}{4}{c}_{3}{({\omega }_{\mu }{\omega }^{\mu })}^{2}\mathrm{.}\end{array}$$

The Lagrangian density includes the interactions between the nucleon field and three meson fields: an isoscalar-scalar *σ*, an isovector-vector ${b}_{0}^{\mu }$ and a vector *ω*_*μ*_, the Coulomb interaction *A*_*μ*_, and the nonlinear self-interactions of meson fields. The meson self-interactions are included to adjust the incompressibility and the stiffness of the EOS in the high-density region. In the RMF approximation, the nuclear EOS including the energy density and the pressure can be derived from the Lagrangian density^60^. The nucleon potential in the Dirac equation reads

2 $${U}_{N}=-{g}_{\sigma N}{\sigma }_{0}+{g}_{\omega N}{\omega }_{0}+{g}_{\rho N}{\tau }_{3}{b}_{0}+e\frac{1+{\tau }_{3}}{2}{A}_{0}\mathrm{.}$$

The Lagrangian of the *K* ^−^ N interaction is given by^44^

3 $${ {\mathcal L} }_{KN}={({{\mathscr{D}}}_{\mu }K)}^{\dagger }({{\mathscr{D}}}^{\mu }K)-({m}_{K}^{2}-{g}_{\sigma K}{m}_{K}\sigma ){K}^{\dagger }K,$$

where the covariant derivative is expressed as

4 $${{\mathscr{D}}}_{\mu }\equiv {\partial }_{\mu }+i{g}_{\omega K}{\omega }_{\mu }+i{g}_{\rho K}{b}_{0\mu }+ie\frac{1+{\tau }_{3}}{2}{A}_{\mu },$$

with the *g*_*ik*_ (*i* = *σ, ω, ρ*) being the corresponding meson-*K* ^−^ coupling constants and *m*_*K*_ being the mass of *K* ^−^ meson. Here, the *K* ^−^N interaction is mediated by *σ* − *K* ^−^, *ω* − *K* ^−^, *ρ* − *K* ^−^ and photon-*K* ^−^ couplings. The resulting Dirac equation for nucleons and Klein-Gordon equation for *K* ^−^ meson, obtained from the Lagrangian in the RMF approximation, are solved in a self-consistently iterative way^45^.

## Results and Discussions

Interestingly, the meson-mediated interactions for *K* ^−^ are coherently attractive, giving rise to the strongly attractive *K* ^−^ N interaction. This is supported by fitting the kaonic atom data^64–69^ or by analyzing the low-energy *K* ^−^ N scattering data based on chirally motivated models^70–75^. From the former, the *K* ^−^ optical potential may reach as deep as −180 MeV at saturation density^65–67^, and from the latter the strong attraction can also be remarkable with a deep depth of −120 MeV^74,75^. It should be noted that the applicability of chirally motivated models should be restrained by the chiral dynamics to low densities. Whereas both methods need some specific extrapolations to saturation density, diversification appears with a much shallower depth around −50 MeV in both methods^64,68,71,72^. Fortunately, the heavy-ion collisions can provide a direct way to extract the *K* ^−^ potential at appropriately produced densities. In the past, various heavy ion collisions got the almost consistent *K* ^−^ potential depth around −100 MeV at saturation density^76,77^. Recently, the direct approval of the strongly attractive *K* ^−^ N interaction was provided one more time by the deep *K* ^−^ optical potential depth, −100 MeV at saturation density^78^, extracted from the collision data from the KaoS Collaboration^79–81^. To investigate the nuclear EOS effects on the nuclear halo, we thus use the *K* ^−^ optical potential depth of −100 MeV at saturation density. Eventually, the effect of various *K* ^−^ optical potential depthes on the halo will be concerned. The coupling constants *g*_*ρK*_ and *g*_*ωK*_ are determined by the SU(3) relation: 2*g*_*ωK*_ = 2*g*_*ρK*_ = *g*_*ρπ*_ = 6.04, while the unique free parameter, *g*_*σK*_, is adjusted to fit the depth of *K* ^−^ optical potential^78^.

**Figure 1:**
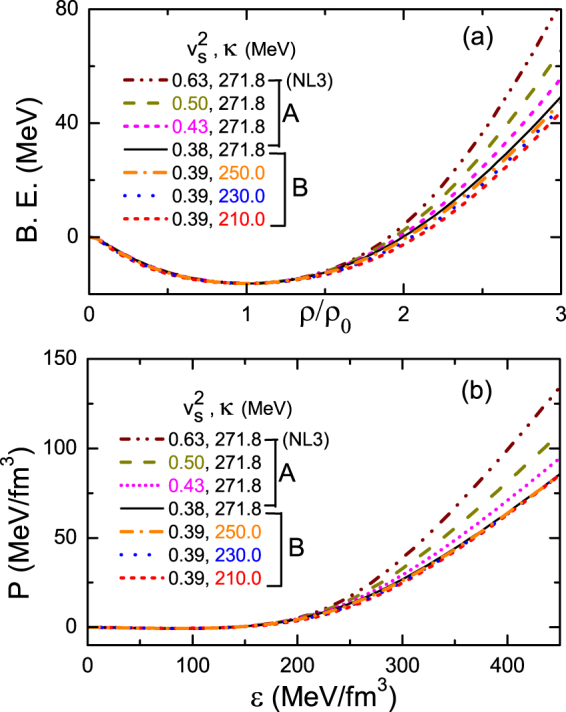
The binding energy per nucleon (B. E.) as a function of nuclear density (upper panel) and the relationship between the pressure and energy density (lower panel) for various EOS’s of symmetric matter. The sound velocity square ${v}_{s}^{2}$ at 2.5*ρ*_0_ and incompressibility *κ* at ${\rho }_{0}(=0.148\,f{m}^{-3})$ are denoted for each curve.

We simulate the uncertainty of the nuclear EOS by adjusting the strengthes of the self-interacting terms in Eq. (1) in a traditional way^59,63^, while the other parameters in ℒ_*int*_ are just moderately modified (less than 5%). As a result, the various stiffness of the EOS is produced at supra-normal densities and different incompressibility is produced at saturation density (*ρ*_0_). Here, we take the famous NL3 parameter set as a starting point^82^, like the parametrization work in ref.^63^. Figure 1 shows different nuclear EOS’s given in two schemes. In Scheme A, we soften the nuclear EOS in the high-density region but keep the saturation property unchanged. Such EOS softening, denoted in Fig. 1 by the descending sound velocity square ${v}_{s}^{2}$ with ${v}_{s}^{2}=\partial P/\partial \varepsilon$ being the partial derivative of the pressure with respect to the energy density, can be realized by increasing the *ω* meson self-interacting coupling *c*_3_ and correspondingly adjusting the parameters of the *σ* meson self-interacting terms. The Scheme B is given to determine a particular incompressibility *κ* at *ρ*_0_ by modifying mainly the parameters of the *σ* meson self-interacting terms while with a fixed *c*_3_ = 60. This yields a series of EOS’s with a similar high-density behavior but with a different incompressibility. These two schemes enable us to investigate separately the effects of the high-density EOS stiffness and of the saturation property on the formation of the nuclear halo. The relevant parameter sets concerning these two schemes are given in Table 1.

**Table 1:** The parameters for various nuclear EOS’s and the *g*_*σK*_ used in Scheme A (upper rows) and Scheme B (lower rows). The unlisted parameters are the same as those of the parameter set NL3. *g*_2_ and *m*_*σ*_ are in unit of *fm* ^−1^ and MeV, respectively. Also given are the sound velocity square ${v}_{s}^{2}$ at 2.5*ρ*_0_ and the incompressibility ***κ*** (in unit of MeV).

Scheme	*c*_3_	*g*_2_	*g*_3_	*g*_*σN*_	*g*_*ωN*_	*g*_*σK*_	*m*_*σ*_	${{\boldsymbol{v}}}_{{\boldsymbol{s}}}^{{\bf{2}}}$	*κ*
A	0	10.431	−28.885	10.217	12.868	1.455	508.2	0.63	271.8
	20	9.833	−23.290	10.274	12.985	1.496	508.2	0.50	271.8
	40	9.205	−17.781	10.323	13.097	1.535	508.2	0.43	271.8
	60	8.604	−12.444	10.370	13.205	1.572	508.2	0.38	271.8
B	60	11.369	−22.987	10.164	13.205	1.541	490.0	0.39	210.0
	60	10.479	−19.702	10.214	13.205	1.548	495.0	0.39	230.0
	60	9.656	−16.472	10.303	13.205	1.562	502.0	0.39	250.0
	60	7.695	−8.875	10.437	13.205	1.582	514.0	0.38	290.0

**Figure 2:**
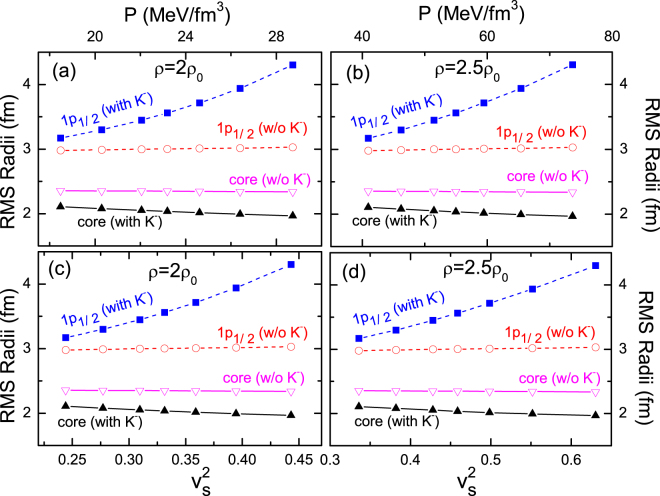
The RMS radii of the core and the outmost layer neutron in ^13^C and ${}_{{K}^{-}}^{13}{\rm{C}}$ as a function of the pressure (up panels) and sound velocity square (lower panels) at *ρ* = 2*ρ*_0_ (left panels) and 2.5*ρ*_0_ (right panels) for various EOS’s in Scheme A. The “w/o *K* ^−^” and “with *K* ^−^” represent normal nuclei and kaonic nuclei respectively.

With the parameter sets that give rise to the nuclear EOS’s of symmetric matter shown in Fig. 1, we investigate the halo phenomenon in light kaonic nuclei that feature the outmost layer nucleons in the 1*p*_1/2_ orbital. Here, we take ^13^C as the seed nucleus for the *K* ^−^ implantation as an example. Figure 2 displays root-mean-square (RMS) radii of the core ^12^C and the 1*p*_1/2_ neutron in ^13^C and ${}_{{K}^{-}}^{13}{\rm{C}}$ as a function of the pressure and the sound velocity square at *ρ* = 2*ρ*_0_ and 2.5*ρ*_0_ which are the densities reachable within the kaonic nuclei, see Table 2. As shown in Fig. 2, the radius of the 1*p*_1/2_ neutron in ${}_{{K}^{-}}^{13}{\rm{C}}$ increases very significantly with the stiffening of the EOS at supra-normal densities, while all other radii including the radius of the 1*p*_1/2_ neutron in normal ^13^C are insensitive to the variation of the high-density EOS. With the stiffening of the high-density EOS, a diffusive neutron halo thus forms in ${}_{{K}^{-}}^{13}{\rm{C}}$, while there is anyway no halo phenomenon in normal ^13^C, in accord with experiments. Meanwhile, as seen from Table 2, the core radius of ${}_{{K}^{-}}^{13}{\rm{C}}$ is clearly smaller than that of normal ^13^C due to the shrinkage induced by the strong *K* ^−^ *N* attraction. Interestingly, the correlation between the radius of the 1*p*_1/2_ halo neutron and the pressure and sound velocity square is nearly linear at supra-normal densities, especially at a higher density. These results provide us a striking perspective to constrain the high-density EOS through the correlation with the low-density halo in the same nutshell, which lowers greatly the large uncertainty of the conventional extrapolation method. Analogously established are the correlations between the radii of the core and outmost layer neutron and the incompressibility at saturation density. We can observe from the data in Table 2 that the radius of the 1*p*_1/2_ halo neutron decreases almost linearly with the rise of the incompressibility.

**Table 2:** The core and 1*p*_1/2_ neutron radii *R*_*c*_ and *R*_*h*_, the maximum nuclear density and the single-neutron binding energies in ${}_{{K}^{-}}^{13}{\rm{C}}$ with various EOS’s. The columns denoted by the ${v}_{s}^{2}$ (at 2.5*ρ*_0_) and *κ* values correspond to Scheme A and B, respectively. The column “^13^C (NL3)” denotes the properties of normal ^13^C obtained with the NL3. The binding energies and radii are in unit of MeV and fm, respectively.

	^13^C (NL3)	${}_{{{\bf{K}}}^{{\boldsymbol{-}}}}^{{\bf{13}}}{\bf{C}}$ (Scheme A)				${}_{{{\bf{K}}}^{{\boldsymbol{-}}}}^{{\bf{13}}}{\bf{C}}$ (Scheme B)				
		${{\boldsymbol{v}}}_{{\boldsymbol{s}}}^{{\bf{2}}}$ = 0.63	${{\boldsymbol{v}}}_{{\boldsymbol{s}}}^{{\bf{2}}}$ = 0.50	${{\boldsymbol{v}}}_{{\boldsymbol{s}}}^{{\bf{2}}}$ = 0.43	${{\boldsymbol{v}}}_{{\boldsymbol{s}}}^{{\bf{2}}}$ = 0.38	*κ* = 290.0	*κ* = 271.8	*κ* = 250.0	*κ* = 230.0	*κ* = 210.0
*R*_*c*_	2.34	1.97	2.02	2.05	2.08	2.10	2.08	2.05	2.03	1.99
*R*_*h*_	3.16	4.30	3.72	3.45	3.30	3.22	3.30	3.41	3.53	3.68
*ρ*_*Max*_/*ρ*_0_	1.53	2.53	2.46	2.41	2.35	2.26	2.35	2.45	2.54	2.62
1*s*_1/2_	43.90	87.10	74.99	68.12	63.99	61.34	63.99	68.25	72.91	79.25
1*p*_3/2_	17.90	25.23	23.83	22.93	22.36	22.01	22.36	22.96	23.59	24.54
1*p*_1/2_	8.45	1.41	2.90	4.12	5.05	5.48	5.05	4.42	3.87	3.21

**Figure 3:**
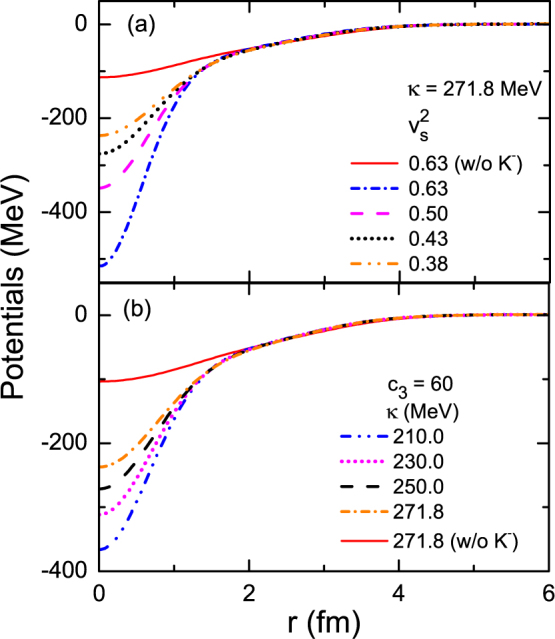
The nucleon potential, given by Eq.(2), as a function of radius with various nuclear EOS’s in ^13^C and ${}_{{K}^{-}}^{13}{\rm{C}}$: (**a**) with the given *κ* = 271.8 MeV (Scheme A), and (**b**) with the given *c*_3_ = 60 (Scheme B).

To reveal the physics behind the sensitive correlations between the EOS and the halo structure in kaonic nuclei, we plot the nucleon potential as a function of radius in Fig. 3. It is seen that the implantation of the *K* ^−^ meson deepens the nuclear potential greatly. The deepening effect is clearly strengthened due to the enhancement of the *K* ^−^-nucleon attraction either through stiffening the EOS at supra-normal densities (with larger ${v}_{s}^{2}$ and resulting stiffer vector field *ω*_0_, panel a) or by ensuring easier compression (with smaller *κ* at *ρ*_0_, panel b). As a consequence, the clear separation in the nucleon potential for various EOS’s yields rather distinct ground-state properties of kaonic nuclei. Indeed, we can see from Table 2 that the separation between neighboring neutron energy levels in ${}_{{K}^{-}}^{13}{\rm{C}}$ becomes progressively large with increasing ${v}_{s}^{2}$ or reducing *κ*. This phenomenon can roughly be understood in a simple quantum model with the harmonic potential where the potential with steeper forms gives rise to larger level separation, since the deep potential well in ${}_{{K}^{-}}^{13}{\rm{C}}$ is roughly in remembrance to the harmonic potential. As the deep potential well also resembles in spirit the infinite deep square potential well, we can have a similar understanding of the large level separation caused by the stiffening of the high-density EOS or the reduction of the incompressibility. In fact, the deepening of the nucleon potential in kaonic nuclei may increase the spin-orbit potential of the 1p orbitals^42,45^, leading to a large spin-orbit splitting that pushes the 1*p*_1/2_ neutron outwards to the continuum. Therefore, the outmost 1*p*_1/2_ neutron in ${}_{{K}^{-}}^{13}{\rm{C}}$ features a small binding energy and a large RMS radius, characterizing the birth of the halo structure. As the incompressibility is constrained progressively well, see^5,83^ and references therein, the stiffness of the high-density EOS should dominate the halo structure. Meanwhile, the core radius of ${}_{{K}^{-}}^{13}{\rm{C}}$ becomes shrunk because of the deep binding of interior states in ${}_{{K}^{-}}^{13}{\rm{C}}$. The shrinkage induced by the strong *K* ^−^ *N* attraction gives rise to a large maximum density that can reach up to 2.62*ρ*_0_. Note that the nucleon potentials with various parameterizations in normal ^13^C are quite similar with each other, consistent with the proximity in their RMS radii and single-particle properties. Besides, the parameterizations with various symmetry energies just have negligible influence on the halo in nuclei of *β*-stability since the isovector potential is small, compared with the deep nucleon potential well.

It is worthy to point out that the sensitive dependence of the halo structure on the nuclear EOS is also discovered in other light kaonic nuclei that feature the nucleon occupation of the outmost 1*p*_1/2_ orbital, e.g., ${}_{{K}^{-}}^{13}{\rm{C}}$, ${}_{{K}^{-}}^{13}{\rm{C}}$, and etc. In addition, we have examined the model dependence of the correlation relationships. The simulation based on the RMF parameter set TM2^84^, rather different from the NL3, indicates that the sensitive correlation between the nuclear EOS and the halo preserves and is rather model independent. Moreover, we find that the conclusions are qualitatively the same with the *K* ^−^ optical potential depth ranging from −80 to −120 MeV which is coincident with the range extracted from the proton-nucleus and nucleus-nucleus collisions^76–81^. Within this range, we have incorporated the effect of *K* ^−^ absorption in nuclei by introducing an imaginary optical potential in a ‘t*ρ*’ form^42^ that associates with the two-body absorption processes ${K}^{-}+N\to Y+\pi ,(Y={\rm{\Lambda }},{\rm{\Sigma }})$. It is found that the width of *K* ^−^ in ${}_{{K}^{-}}^{13}{\rm{C}}$ is around 45 MeV. In this circumstance, the imaginary part of *K* ^−^ optical potential just has small effects on the stationary state properties of kaonic nuclei and is neglected in the numerical formulation^42,45,85^.

A vital issue for the kaonic nuclei is the experimental production. There is still no experiment that identifies the production of the kaonic nuclei. We should note that a *K* ^−^ width of tens of MeV in kaonic nuclei gives a characteristic lifetime of the strong interaction, 10^−23^ s. Such a short time makes the detection of kaonic nuclei rather difficult^46,48,49,52,53^. A very recent research aimed to address this experimental fact by introducing a nonlinear form (in *ρ* ^2^ or *ρ* ^3^), associated with the *K* ^−^ multinucleon interactions, to modify the optical potential beyond the ‘*tρ*’ approximation based on chirally motivated models, and gave the possibility of a large *K* ^−^ width that may elaborate the experiments for the undiscovered kaonic nuclei^86^. Though the result is interesting due to the modification to the ‘*tp*’ approximation, the specific nonlinear form and its parameters are not unique. As the intermediate metastable state is included, the kaon optical potential can return to the nearly linear form at higher densities^87^. This result may have an implication to the mechanism of the intermediate state plus a spectator which reflects a two-body feature of the multinucleon interactions. Since the conclusion on the existence of kaonic nuclei in Ref.^86^ relies on the specific form of the *K* ^−^ multinucleon attribution, it is difficult to reach a conclusive exclusion to the existence of kaonic nuclei^40,41,46,48,49,52,53^. In fact, the theoretical description of the interaction between kaons and nucleons is greatly dependent on physical models, energy and density^65,66,69–74,87,88^, which makes the prediction of kaonic nuclei quite different. In this work, we use the RMF models to study the static properties of kaonic nuclei, based on the fact that the RMF approximation works better for denser matter due to the blocking of the contribution of the intermediate states. This primary merit of the RMF models makes us believe that the study of kaonic nuclei is rather optimistic. Anyway, the existence of kaonic nuclei is still under intense debate^40,46–49,52,53,86^ and needs the clarification via future experiments. As an experimental suggestion, we propose that the photo-nucleus or pion-nucleus reaction^51,89^, e.g., *γ* + ^13^C $\to$ *K* ^+^ + ${}_{{K}^{-}}^{13}{\rm{C}}$ or ${\pi }^{-}$ + ^13^ *N* $\to$ *K* ^*+^ + ${}_{{K}^{-}}^{13}{\rm{C}}$, may be used to produce kaonic nuclei, with an anticipation of the halo radius measurement via the correlation of the outgoing kaons with the halo neutron due to the strong interaction. We would hope that the light kaonic nuclei can be used as the favorable candidates to constrain the nuclear EOS at supra-normal densities posterior to performing the experiments.

## Summary

In this work, we have investigated in the RMF theory the novel halo formation due to the strong *K* ^−^ *N* attraction in light kaonic nuclei that feature the outmost nucleons in the 1*p*_1/2_ orbital. It is found that the low-density halo radius correlates very sensitively with the nuclear EOS of symmetric matter at saturation density and in the region of supra-normal densities which could form in the core of light kaonic nuclei. Facing up large uncertainties of the nuclear EOS at supra-normal densities either due to extrapolations with any nuclear model or from the extractions through heavy-ion reactions or celestial observations of neutron stars, the present method with the structural exploration has the appealing merit to evade from such uncertainties. In particular, the determination of the high-density EOS can be implemented through the nearly linear correlation between the property of low-density diffusive halos and the pressure and sound velocity square in the region with a density up to 2.5*ρ*_0_. We hope that the present study may urge more theoretical explorations and especially the timely experiments for light kaonic nuclei.
